# Recognition Pattern of the *Fasciola hepatica* Excretome/Secretome during the Course of an Experimental Infection in Sheep by 2D Immunoproteomics

**DOI:** 10.3390/pathogens10060725

**Published:** 2021-06-09

**Authors:** David Becerro-Recio, Javier González-Miguel, Alberto Ucero, Javier Sotillo, Álvaro Martínez-Moreno, José Pérez-Arévalo, Krystyna Cwiklinski, John P. Dalton, Mar Siles-Lucas

**Affiliations:** 1 Parasitology Unit, Institute of Natural Resources and Agrobiology of Salamanca (IRNASA-CSIC), 37008 Salamanca, Spain; david.becerro@irnasa.csic.es (D.B.-R.); albertucero@mncn.csic.es (A.U.); mmar.siles@irnasa.csic.es (M.S.-L.); 2 Department of Evolutionary Ecology, National Museum of Natural Sciences (MNCN-CSIC), 28006 Madrid, Spain; 3 National Centre for Microbiology, Instituto de Salud Carlos III, Majadahonda, 28220 Madrid, Spain; javier.sotillo@isciii.es; 4 School of Veterinary Medicine, University of Cordoba, 14014 Cordoba, Spain; amm@uco.es (Á.M.-M.); an1pearj@uco.es (J.P.-A.); 5 Molecular Parasitology Laboratory, Center of One Health (COH) and Ryan Institute, School of Natural Science, National University of Ireland Galway, H91 DK59 Galway, Ireland; krystyna.cwiklinski@nuigalway.ie (K.C.); johnpius.dalton@nuigalway.ie (J.P.D.)

**Keywords:** *Fasciola hepatica*, sheep, excretory/secretory antigens, immunoproteomics

## Abstract

Excretory/secretory products released by helminth parasites have been widely studied for their diagnostic utility, immunomodulatory properties, as well as for their use as vaccines. Due to their location at the host/parasite interface, the characterization of parasite secretions is important to unravel the molecular interactions governing the relationships between helminth parasites and their hosts. In this study, the excretory/secretory products from adult worms of the trematode *Fasciola hepatica* (FhES) were employed in a combination of two-dimensional electrophoresis, immunoblot and mass spectrometry, to analyze the immune response elicited in sheep during the course of an experimental infection. Ten different immunogenic proteins from FhES recognized by serum samples from infected sheep at 4, 8, and/or 12 weeks post-infection were identified. Among these, different isoforms of cathepsin L and B, peroxiredoxin, calmodulin, or glutathione S-transferase were recognized from the beginning to the end of the experimental infection, suggesting their potential role as immunomodulatory antigens. Furthermore, four FhES proteins (C2H2-type domain-containing protein, ferritin, superoxide dismutase, and globin-3) were identified for the first time as non-immunogenic proteins. These results may help to further understand host/parasite relationships in fasciolosis, and to identify potential diagnostic molecules and drug target candidates of *F. hepatica*.

## 1. Introduction

Fasciolosis is a foodborne trematodosis caused by platyhelminthes from the family Fasciolidae, primarily represented by the species *Fasciola hepatica* and *F. gigantica* that have a worldwide distribution as a result of their ability to develop in a variety of habitats and hosts [1]. Infection in the definitive host begins following ingestion of a number of *Fasciola* metacercariae, which excyst in the small intestine releasing the newly excysted juveniles (NEJ). The NEJ migrate through the tissues until they reach the liver between 4 and 6 days after infection. Once there, *Fasciola* juveniles burrow through the liver [~4th week post-infection (PI)] and remain in the liver parenchyma until the parasite performs a final migration to the bile ducts, an immunologically favorable environment, in which it grows into an adult and reaches reproductive maturity (~8th week PI). Here, the parasite begins shedding its eggs that reach the intestinal lumen, which are expelled with the feces (~12th PI). This habitat allows *Fasciola* to survive for long periods, which have been reported up to 13 years [2,3,4]. The initial migration represents the acute phase of the infection, which is characterized by internal liver lesions, hemorrhage and a variety of cellular and humoral inflammatory responses [5,6]. The chronic phase of the infection comprises the stage of the parasite in the bile ducts and corresponds with anemia and weight loss [7], together with changes in blood parameters [8]. However, in most cases of infection in sheep and cattle the parasitic burden is low, and the disease remains asymptomatic [9].

Fasciolosis has a detrimental effect on animal health and production causing economic losses that are estimated over 3$ billion per year [10], although recent estimations conclude that these costs can be significantly much higher [11]. Apart from its importance in agriculture, fasciolosis affects at least 2.4 million people, and up to 90 million people are thought to be at risk of infection [12,13]. Nevertheless, the lack of molecular detection methods in developing countries and the frequent subclinical nature of the disease makes it difficult to track the disease epidemiology and development and to offer reliable data. The diagnosis of *Fasciola* infection has been performed traditionally through the counting of eggs in feces. Due to the limitations of this technique [14,15], molecular alternatives, such as ELISA and RT-PCR, have been developed aiming to achieve higher sensitivity and allow early detection [16,17,18].

Although triclabendazole has proved to be effective against the juvenile and adult forms of *Fasciola* [19], there are important concerns surrounding the use of chemical treatments; these include issues such as the appearance of drug resistant parasites [20,21], the presence of the drug and metabolites in milk [22], or the inability to provide long term protection as animal become re-infected as soon as the drug is eliminated. For this reason, vaccines are considered as a desirable alternative and trials using different approaches have been performed [23,24,25]. However, the assays up to date have achieved limited effectiveness [26].

Little is known about the evolution of the immune response against *Fasciola* during the infection course, therefore, further research is still needed. In this study, sheep were experimentally infected with *F. hepatica*, and serum samples were taken at three different times PI, corresponding to the key moments in *Fasciola* development (4th, 8th, and 12th weeks PI). The excretory/secretory products from adult worms of *F. hepatica* (FhES) were separated by two-dimensional polyacrylamide gel electrophoresis (2D-PAGE), transferred to Western blot membranes and incubated with the serum samples in order to identify immunogenic proteins by mass spectrometry (MS). This study expands our understanding of how ruminant immune recognition changes during the course of *F. hepatica* infection.

## 2. Results and Discussion

In order to evade the host immune response, helminth parasites release excretory/secretory (ES) products as a finely regulated mechanism that facilitates host immunomodulation and that usually leads to chronic infections [27]. This could be considered to be the case during infection with *F. hepatica*, which is responsible for chronic infections in ruminants. As a consequence of a parasite-driven mixed Th1/Th2 response that then shifts to a Th2-type cytokine pattern devoid of the inflammatory component the parasite is able to reach its adult stage [28]. *F. hepatica* secretions have been widely studied in the last few years due to the application of the new -omics techniques [29]. Thus, this antigenic compartment has been described as a complex mixture of not only ES products, but also tegument-derived molecules as well as its derived extracellular vesicles [30]. Although both the composition and immunomodulatory properties of these antigens are known, there is a lack of knowledge regarding their immunogenic recognition during the progress of the fasciolosis infection. The main objective of this study was to evaluate the immunogenic pattern of the FhES antigens during the course of an experimental infection in sheep. For this purpose, 36 serum samples from a total of 9 sheep were pooled according to 4 previously established conditions (preimmune sera as negative controls and sera taken at 4-, 8-, and 12-weeks PI). The time-points were chosen for their coincidence with relevant periods in the development of *F. hepatica* in the vertebrate host. 

**Figure 1:**
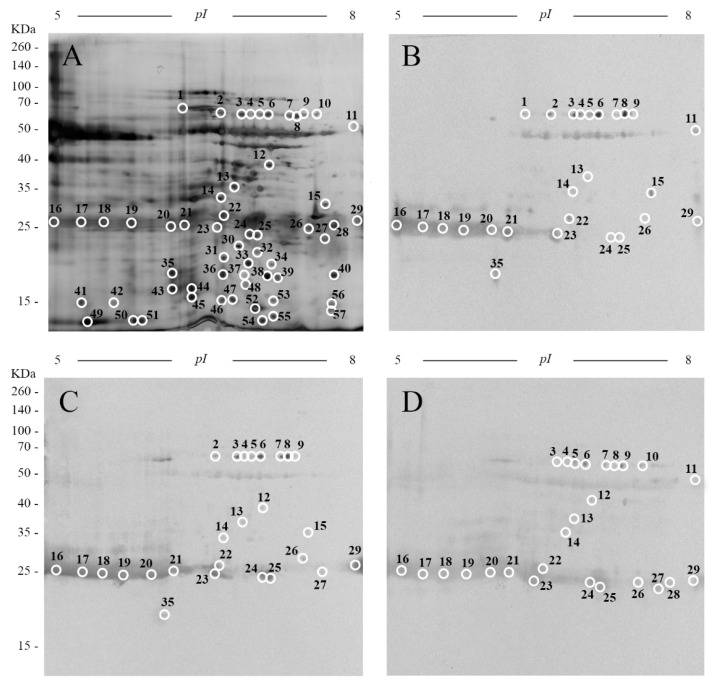
(**A**) Representative two-dimensional electrophoresis of 40 µg of the excretory/secretory products from adult worms of *F. hepatica* (FhES). The gels were in the 5–8 isoelectrical point range, 12% polyacrylamide and silver-stained, and Western blots showing the immunogenic spots of the FhES extract revealed by pools of serum samples from experimentally infected sheep collected at (**B**) 4, (**C**) 8, and (**D**) 12 weeks post-infection. Reference molecular weight masses are indicated on the left. The spots analyzed by mass spectrometry are circled and numbered.

To obtain an overall view of all the proteins of the FhES, this preparation was analyzed by 2D-PAGE. Proteins were firstly electrofocused using 3–10 linear immobilized pH gradient strips. Silver nitrate staining of these 2D gels revealed about 410 spots in the excretome/secretome of *F. hepatica*, many sparsely distributed across isoelectric points (pIs) between 5 and 9.8, and a broad range of molecular weights (MWs) (10–170 kDa). Only 12 spots were observed in the 2D gels with pIs < 5 (Figure S1). In order to improve spot resolution and detection, the FhES were electrofocused in 5–8 IPG strips. With this new condition, silver staining revealed a total of 448 spots in the excretome/secretome of *F. hepatica* over a broad range of MWs (10–170 kDa) (Figure 1A). 

Secondly, the evolution of the recognition pattern during the course of *F. hepatica* infection was studied by immunoblot analysis carried out with the above-mentioned serum samples from sheep. Analysis of the 2D membranes allowed the localization of 86, 95, and 115 immunogenic spots at 4-, 8-, and 12-weeks PI, respectively (Figure 1B–D). This represents recognition rates of 19.2, 21.2 and 25.7% from the total observed spots in the 2D gels. Immunogenic spots showed a wide range of pIs (between 5 and 8), while their MWs were located within a narrower range (between 20 and 70 kDa). Serum samples from experimentally infected sheep before infection (control) only showed nonspecific binding of the secondary antibody to be negligible in the 50 kDa band (11 spots, which were not considered for further analysis) (Figure S2).

The selected major spots localized by matching with those recognized by experimentally infected sheep (n = 30) and additionally those non-immunogenic (n = 27) were manually excised from 2D gels and submitted to analysis by MS. Up to 15 of the 57 analyzed spots (26.31%) were identified. Among them, between 1 and 3 different proteins from each spot, and between 1 and 6 isoforms of the same protein were identified, corresponding to 14 different proteins. It is worth mentioning that despite the fact that the parasite genome has been recently published [31] and major advances have been made through -omics technologies in the study of *F. hepatica* proteomic composition [29], the existence of available annotation information on *F. hepatica* proteins in the databases is still scarce. Thereby, this fact could explain, together with the high stringency used in the database search, the low percentage of identification reached in this study. Table 1 shows the description of the identified proteins, the accession to Uniprot database (obtained after homology analysis at Uniprot database), the sequence coverage, as well as the molecular function and biological processes in which the proteins identified are involved according to GO analysis (complete dataset of the MS results is shown in Table S1). In addition, Table 1 shows the time PI in which each identified spot is recognized by the serum samples from experimentally infected sheep. The majority of immunogenic identified spots (7/11) were recognized at all three studied time points (4-, 8-, and 12-weeks PI). These included different isoforms of cathepsins B and L, peroxiredoxin, glutathione S-transferase, as well as fimbrin, calmodulin, and a proteasome subunit alpha type. 

Cathepsins represent the most abundant products found in the ES antigenic compartment of *F. hepatica* [32]. This family of papain-like cysteine peptidases has been widely studied in the field of fasciolosis due to their pivotal role in important processes such as host hemoglobin digestion for nutrition, invasion, migration, or plasminogen-binding processes [33,34]. Peroxiredoxin and glutathione S-transferase belong to important super-families with protective functions against oxidative stress [35,36]. Specifically, peroxiredoxin is an antioxidant of the secretions of *F. hepatica*, which apart from its peroxidase protective role, it has been also linked with the recruitment of alternatively activated macrophages to the peritoneum and thus, with the induction of a polarized Th2 response [37]. In addition, a number of isoforms of the glutathione S-transferase family have been previously identified in *F. hepatica* accounting for as much as 4% of the parasite secretions and developing detoxification functions [38]. The fact that these proteins were recognized from the beginning to the end of the experiment in our study suggests their immunomodulatory role within the vertebrate host–parasite relationships, as it has been recently reviewed by Ryan et al. [28]. In this context, different *F. hepatica* secreted antigens belonging to these families such as cathepsin B and L, peroxiredoxin and glutathione S-transferase have been postulated as vaccine candidates, which show variability in vaccine efficacy between trials [23,26]. As shown by this study, these proteins are represented by various isoforms within the *F. hepatica* secretome, suggesting the presence of redundancy mechanisms within their biochemical systems that could make the deleterious effects of the immune response induced by vaccination with a defined antigen more difficult to be reached [4]. Thereby, other immunogenic proteins identified in this study with apparently less isoforms or that are less represented within the parasite secretions, such as fimbrin, calmodulin, and proteasome subunit alpha type could represent new promising targets for fasciolosis control.

In addition, isoforms of the epididymal secretory protein E1 and peroxiredoxin were recognized at 4- and 8- weeks, and at 8- and 12-weeks PI, respectively. More specifically, thioredoxin glutathione reductase and lysosomal Pro-X carboxypeptidase were only recognized at 12 weeks PI. Future research could be conducted to confirm that all isoforms belonging to these proteins are specifically recognized at this time point. Therefore, these antigens could be used as diagnostic tools for a specific moment of the disease. Regrettably, no proteins were identified being specifically recognized at earlier stages of the infection (4 weeks PI), which would have been important from the point of view of diagnosis in a period in which *F. hepatica* has reached neither its final location nor its adult stage.

Finally, four proteins (C2H2-type domain-containing protein, ferritin, superoxide dismutase (SOD), and globin-3) and an isoform of the epididymal secretory protein E1 were identified as non-immunogenic antigens. These proteins could be of paramount importance, since despite being part of the parasitic secretions that are released to the host–parasite interface, do not trigger an immune response and could be related to parasite evasion mechanisms. Furthermore, as these proteins are not recognized by the host immune system, their associated biological processes could be of vital importance for the parasite development. In this context, in order to reach its adult stage, *F. hepatica* must enter the bile duct and feed on blood [39]. As a consequence, iron and oxygen compounds might be required not only for parasite nutrition, but also for egg production in line with other reports on schistosomes [40]. For that reason, parasitic antigens such as ferritins and globins with functions on iron and oxygen transport and metabolism, respectively, could be of importance within the *Fasciola* life cycle.

Globins are members of a broad group of oxygen-transport proteins, although their role as extracellular antigens is currently a matter of debate [41]. Globins functions might be specially striking as part of antigenic extracts from the host–parasite interface of trematodes. These parasites usually live in semi-anaerobic environments, so extracellular globins may have biological functions beyond oxygen transport and storage. Among them, oxygen scavenging, heme reserve for egg production, and nitric oxide dioxygenase have been postulated [42].

Ferritins are proteins essential for iron balance in eukaryote cells, to which different functions have been attributed, including oxygen transport and detoxification, *F. hepatica* adult worms feed on blood and ferritins could serve to regulate iron balance in the parasite. Several isoforms of ferritin have been described in *F. hepatica*; the Ferritin 1 isoform (FhFtn1) as candidate antigen for the detection of specific antibodies in infected animals [43]. Superoxide dismutases are also related to detoxification, since they have been described as antioxidant enzymes in *F. hepatica* and found already in the secretions of adult worms (e.g., [44]). Here, both the identified ferritin and SOD isoforms were not recognized by infected sheep at any time PI. Importantly, an isoform of the epididymal secretory protein E1 was not immunogenic for infected sheep, while a second isoform was immunogenic, as shown here. This suggest that the immune recognition of specific molecules important for parasite survival depends on the isoform of the protein. Thus, some isoforms are detected suggesting specific antibodies are triggered in infected sheep, but those specific antibodies do not react with other isoforms of the same protein family. This could constitute an evasion mechanism of *F. hepatica*: while some of the isoforms could be “immunogenic screens”, some others avoid antibody recognition and retain their ability to function.

Finally, the overall pattern of proteins of FhES identified in this study correlates with previous similar studies (e.g., [45,46]). Nevertheless, it should be mentioned that the observed immunogenic profile could be qualitatively different in naturally infected animals, which usually tend to have chronic infections from repeated exposure [46]. For that reason, the obtained results should be reassessed in natural infections in relation to their potential practical implication. 

In summary, we have identified a set of *F. hepatica* proteins triggering antibody responses during the course of an experimental infection in sheep, showing that the immune recognition pattern is qualitatively very similar from the 4th to the 12th week PI, with few exceptions of molecules detected only at late time PI (12 weeks PI). We have also shown that prominent spots of proteins present in the secretions of adult worms do not elicit antibody responses at any time PI of those assessed here and that inside a family of parasite proteins, some isoforms trigger antibody responses, while some other isoforms do not elicit detectable antibodies in infected animals. Among those which are not recognized, some could have the potential to induce a protective immune response against *F. hepatica* infection after vaccination.

## 3. Materials and Methods

### 3.1. Collection of a F. hepatica ES Extract

Adult liver flukes were collected from the livers of Texel-cross sheep 16 weeks after oral infection with 150 *F. hepatica* metacercariae (South Gloucester isolate: Ridgeway Research Ltd., St Briavels, UK), carried out at Moredun Scientific, UK under license from the UK Home Office by the Animal (Scientific Procedures) Act 1986 (License No. PPL/60/4426) after ethical review by the Moredun Scientific Animal Ethics Committee. The parasites were washed with sterile phosphate-buffered saline (PBS) before culturing in RPMI medium (containing 0.1% glucose, 100 U penicillin and 100 mg/mL streptomycin) at a ratio of 1 worm/2 mL at 37 °C and 5% CO_2_. After 2 h, culture media (containing the FhES) was collected and centrifuged at 300 × *g* for 10 min and then 700 × *g* for 30 min to eliminate large debris and frozen at −80 °C prior to use.

### 3.2. Obtention of Sheep Serum Samples at Different Infection Times

Nine eight-month-old male Merino-breed sheep, obtained from a liver fluke-free farm, were used for this study. All animals were tested monthly for parasite eggs by fecal sedimentation, with negative results in all cases. Prior to challenge, all animals were tested for serum IgG specific for *F. hepatica* cathepsin L1 (FhCL1) by an enzyme-linked immunosorbent assay (ELISA), with negative results in all cases. The sheep were housed indoors (100 m^2^ covered and 100 m^2^ uncovered facility) and fed hay and pellets and given water ad libitum. The sheep were orally infected with one dose of 150 metacercariae of the South Gloucester isolate of *F. hepatica* (Ridgeway Research Ltd., St Briavels, UK). Serum samples were obtained just before challenge and at week 4, 8 and 12 PI. The experiment was approved by the Bioethics Committee of the University of Cordoba (code No. 1118) and conducted in accordance with European (2010/63/UE) and Spanish (RD 1201/2005) directives on animal experimentation. 

### 3.3. Protein Separation by 2D-PAGE

To obtain an overall view of all proteins of the FhES, these were separated by 2D-PAGE according to the methods described by González-Miguel et al. [34] with minor modifications. Firstly, FhES was cleaned and purified using the ReadyPrep 2D-CleanUp kit (Bio-Rad, Hercules, CA, USA) following the instructions given by the supplier. The resulting protein samples were then resuspended in rehydration buffer (7M urea, 2M thiourea, 4% 3-[(3-cholamidopropyl) dimethylammonio]-1-propanesulfonate (CHAPS)) for the next step. The samples were divided into 125 μL aliquots (containing 40 μg of protein) and stored at –20 °C until use. When they were used FhES aliquots were supplemented with 50 mM DTT and 0.4% pH 3–10 or 5–8 ampholytes for 15 min, incubated and centrifuged to remove all particulate material, and then applied to 7-cm IPG strips (Bio-Rad, Hercules, CA, USA) with linear pH ranges of 3–10 or 5–8 into a rehydration plate. After 10 min, the strips were covered with mineral oil and incubated at 20 °C for at least 16 h. Once the protein extracts had been fully absorbed by the IPG strips, these were placed in a Protean IEF Cell (Bio-Rad, Hercules, CA, USA) for isoelectric focusing (IEF) for a total of 20,000 Vh. When the process had concluded, strips were reduced and alkylated with 150 mM DTT and 150 mM 2-iodoacetamide in 2D equilibration buffer (urea 6M, 2% SDS, 1.5M Tris/HCl, 30% glycerol), and transferred to a 12% SDS-PAGE gel in which proteins were separated according to their MW. After the second dimension was performed, gels were then silver stained with the PlusOne Silver Staining Kit, Protein (GE Healthcare, Chicago, IL, USA) or transferred to nitrocellulose membranes for their immunoblot analysis. 

### 3.4. Immunoblot Assays

In order to determine the FhES recognition pattern during the course of an experimental infection in sheep, 2D gels were electrotransferred to nitrocellulose membranes at 20 V for 30 min using a Semi-Dry Transfer Cell (Bio-Rad, Hercules, CA, USA). Immunoblots were performed with a total of 36 sheep serum samples taken from the different conditions, which were pooled according to the 4 studied conditions (pre-immune, 4th week PI, 8th week PI, 12th week PI). After transference, membranes were blocked with 2% BSA in PBS-Tween (NaH_2_PO_4_ 2.5 mM, Na_2_HPO_4_ 7.5 mM, NaCl 145 mM, Tween_20_ 0.05%) overnight at 4 °C, and incubated with a 1:200 dilution of each pool for 1 h at 37 °C. After 3 × 5 min washes with PBS-Tween_20_, a 1:4000 dilution of a horseradish peroxidase-labelled anti sheep IgG was added for 1 h at 37 °C. Finally, the membranes were revealed using 4-chloro-1-naphtol 0.05%. The samples were analyzed in triplicate to assess the overall reproducibility of the protein and immunogen spots patterns and only minor differences were observed between gel replicates.

### 3.5. Image Acquisition and Spot Selection

The 2D silver-stained gels together with the nitrocellulose membranes were scanned using the ChemiDoc MP Imaging System (Bio-Rad, Hercules, CA, USA). The 2D images of immunoblots and their homologous silver-stained gels were aligned to Ips and MWs and then matched with the PDQuest Software v.8.0.1 (Bio-Rad, Hercules, CA, USA) to identify the immunogenic spots in the gels. 

### 3.6. Spot Identification by Mass Spectrometry

A major representation of both the immunogenic spots that could be matched with the 2D gels, as well as the non-immunogenic spots, were selected and excised from the 2D silver-stained gels using a 1000 µL sterile pipette tip and placed in a 1.5 mL tube containing 500 µL of ultrapure water. Protein identification was performed at the proteomics facility of SCSIE University of Valencia (Valencia, Spain) for MS analysis. The samples were digested with sequencing grade trypsin (Promega, Madison, WI, USA) as previously described [47]. The digestion was stopped with 1% trifluoroacetic acid and the digested peptides were concentrated. A BSA plug was analyzed in the same way to control the digestion process.

Digested spots were resuspended and 5 µL loaded onto a trap column (NanoLC Column, 3 µ C18-CL, 350 um × 0.5 mm; Eksigent) and desalted with 0.1% trifluoroacetic acid at 3 µL/min during 5 min. Peptides were then loaded onto an analytical column (LC Column, 3 µ C18-CL, 75 um × 12 cm, Nikkyo) equilibrated in 5% acetonitrile and 0.1% formic acid. Elution was carried out with a linear 5–45% gradient of solvent B (95% acetonitrile, 0.1% formic acid) at a flow rate of 300 nL/min. and peptides were analyzed in a mass spectrometer nanoESI-Q-TOF (5600 TripleTOF, ABSciex, Framingham, MA, USA). The TripleTOF was operated in information-dependent acquisition mode, in which a 0.25-s TOF MS scan from 350–1250 m/z, was performed, followed by 0.05-s product ion scans from 100–1500 m/z on the 50 most intense 2–5 charged ions. A database search was performed on the *F. hepatica* WormBase ParaSite database (BioProject PRJEB25283 and version WBPS13) concatenated to the cRAP contaminant database (https://www.thegpm.org/crap/) (accessed on 20 May 2019) using Carbamidomethylation of C as fixed modification and deamidation of N and Q and oxidation of M as variable modifications. Precursor and fragment mass tolerance was set to 20 ppm and 0.3 Da, respectively. Only spots matching to two or more validated peptides and with high reliability (>90% confidence), were considered as positive identifications. The molecular function and biological processes of the identified proteins were assigned according to the gene ontology database (http://www.geneontology.org) (accessed on 10 June 2019) and the Swiss-Prot/UniProt database (http://beta.uniprot.org) (accessed on 10 June 2019).

## Figures and Tables

**Table 1:** Immunogenic and non-immunogenic protein spots of FhES extract recognized by pools of sera from experimentally infected sheep with *F. hepatica* and identified by mass spectrometry. Molecular function and biological process in which proteins of FhES extract are involved was assigned according to the Gene Ontology and Swiss-Prot/UniProt databases.

Spot Number	Accession Code	Protein Definition	Sequence Coverage (%)	Molecular Function	Biological Process	Recognition Time (Weeks PI)
10	A8E0R8	Thioredoxin glutathione reductase	22.68	oxidoreductase	cell redox homeostasis	12
	A0A4E0R242	Lysosomal Pro-X carboxypeptidase	10.03	serine-type peptidase	-	12
14	A7UNB2	Cathepsin B	10.5	cysteine-type endopeptidase	regulation of catalytic activity	4–8–12
16	Q24940	Cathepsin L	55.95	cysteine-type endopeptidase	proteolysis	4–8–12
	A0A4E0RY97	Fimbrin, putative	4.23	actin filament binding	actin filament bundle assembly	4–8–12
19	Q24940	Cathepsin L	56.75	cysteine-type endopeptidase	proteolysis	4–8–12
23	Q24940	Cathepsin L	46.43	cysteine-type endopeptidase	proteolysis	4–8–12
	P91883	Peroxiredoxin	16.05	oxidoreductase	cell redox homeostasis	4–8–12
24	Q24940	Cathepsin L	12.5	cysteine-type endopeptidase	proteolysis	4–8–12
	P91883	Peroxiredoxin	11.52	oxidoreductase	cell redox homeostasis	4–8–12
	A4V9Q5	Calmodulin	19.46	calcium ion binding	calcium-mediated signaling	4–8–12
25	E3UTT4	Glutathione S-transferase	36.19	transferase	-	4–8–12
	P91883	Peroxiredoxin	29.89	oxidoreductase	cell redox homeostasis	4–8–12
26	E3UTT4	Glutathione S-transferase	20.9	transferase	-	4–8–12
	P91883	Peroxiredoxin	22.63	oxidoreductase	cell redox homeostasis	4–8–12
	A0A2H1BSW4	Proteasome subunit alpha type	27.66	threonine-type endopeptidase	ubiquitin-dependent protein catabolic process	4–8–12
27	P91883	Peroxiredoxin	39.92	oxidoreductase	cell redox homeostasis	8–12
28	Q24940	Cathepsin L	12.5	cysteine-type endopeptidase	proteolysis	12
	P91883	Peroxiredoxin	16.46	oxidoreductase	cell redox homeostasis	12
33	A0A4E0R9N4	C2H2-type domain-containing protein	7.96	nucleic acid binding	regulation of transcription	No recognition
35	A0A4E0RV44	Epididymal secretory protein E1	10.82	-	intracellular cholesterol transport	4–8
44	A0A4E0RV44	Epididymal secretory protein E1	7.89	-	intracellular cholesterol transport	No recognition
	A0A4E0RD35	Ferritin	21.05	ferric iron binding	cellular iron ion homeostasis	No recognition
47	Q9XY94	Superoxide dismutase [Cu-Zn]	33.77	oxidoreductase	removal of superoxide radicals	No recognition
55	A0A2H1CJ88	Globin-3	9.59	heme binding	oxygen transport	No recognition

## Data Availability

Not applicable.

## Supplementary Materials

The following are available online at https://www.mdpi.com/article/10.3390/pathogens10060725/s1, Figure S1: Representative two-dimensional electrophoresis of 40 µg of the FhES in the 3–10 isoelectrical point range, Figure S2: Western blots of the FhES extract revealed by pool of serum samples from experimentally infected sheep before infection (control), Table S1: Complete dataset of the MS results.

## References

[1] Siles-Lucas M., Becerro-Recio D., Serrat J., González-Miguel J. (2021). Fascioliasis and fasciolopsiasis: Current knowledge and future trends. Res. Vet. Sci..

[2] Mas-Coma S., Bargues M.D., Esteban J.G., Dalton J.P. (1999). Human fasciolosis. Fasciolosis.

[3] Moazeni M., Ahmadi A. (2016). Controversial aspects of the life cycle of *Fasciola hepatica*. Exp. Parasitol..

[4] González-Miguel J., Becerro-Recio D., Siles-Lucas M. (2021). Insights into *Fasciola hepatica* Juveniles: Crossing the Fasciolosis Rubicon. Trends Parasitol..

[5] Harrington D., Lamberton P.H.L., McGregor A. (2017). Human liver flukes. Lancet Gastroenterol. Hepatol..

[6] Mas-Coma S., Valero M.A., Bargues M.D. (2019). Fascioliasis. Adv. Exp. Med. Biol..

[7] Behm C.A., Sangster N.C., Dalton J.P. (1999). Pathology, pathophysiology and clinical aspects. Fasciolosis.

[8] Madhumitha R., Gohel S., Vishwanathan L., Gopalakrishnan R. (2015). Liver Lesions, Fever and Eosinophilia Caused by *Fasciola hepatica* in a 15-year-old Girl. Indian J. Pediatr..

[9] Arslan F., Batirel A., Samasti M., Tabak F., Mert A., Özer S. (2012). Fascioliasis: 3 cases with three different clinical presentations. Turk. J. Gastroenterol..

[10] Alvarez Rojas C.A., Jex A.R., Gasser R.B., Scheerlinck J.P. (2014). Techniques for the diagnosis of *Fasciola* infections in animals: Room for improvement. Adv. Parasitol..

[11] Mehmood K., Zhang H., Sabir A.J., Abbas R.Z., Ijaz M., Durrani A.Z., Saleem M.H., Ur Rehman M., Iqbal M.K., Wang Y. (2017). A review on epidemiology, global prevalence and economical losses of fasciolosis in ruminants. Microb. Pathog..

[12] Keiser J., Utzinger J. (2005). Emerging foodborne trematodiasis. Emerg. Infect. Dis..

[13] Fürst T., Keiser J., Utzinger J. (2012). Global burden of human food-borne trematodiasis: A systematic review and meta-analysis. Lancet Infect. Dis..

[14] Mas-Coma S., Bargues M.D., Valero M.A. (2014). Diagnosis of human fascioliasis by stool and blood techniques: Update for the present global scenario. Parasitology.

[15] Radfar M.H., Nourollahi-Fard S.R., Mohammadyari N. (2015). Bovine fasciolosis: Prevalence, relationship between faecal egg count and worm burden and its economic impact due to liver condemnation at Rudsar abattoir, Northern Iran. J. Parasit. Dis..

[16] Cwiklinski K., O’Neill S.M., Donnelly S., Dalton J.P. (2016). A prospective view of animal and human Fasciolosis. Parasite Immunol..

[17] Sarkari B., Khabisi S.A. (2017). Immunodiagnosis of Human Fascioliasis: An Update of Concepts and Performances of the Serological Assays. J. Clin. Diagn. Res..

[18] Webb C.M., Cabada M.M. (2018). Recent developments in the epidemiology, diagnosis, and treatment of *Fasciola* infection. Curr. Opin. Infect. Dis..

[19] Keiser J., Engels D., Büscher G., Utzinger J. (2005). Triclabendazole for the treatment of fascioliasis and paragonimiasis. Expert. Opin. Investig. Drugs..

[20] Brennan G.P., Fairweather I., Trudgett A., Hoey E., McCoy, McConville M., Meaney M., Robinson M., McFerran N., Ryan L. (2007). Understanding triclabendazole resistance. Exp. Mol. Pathol..

[21] Kamaludeen J., Graham-Brown J., Stephens N., Miller J., Howell A., Beesley N.J., Hodgkinson J., Learmount J., Williams D. (2019). Lack of efficacy of triclabendazole against *Fasciola hepatica* is present on sheep farms in three regions of England, and Wales. Vet. Rec..

[22] Power C., Danaher M., Sayers R., O’Brien B., Clancy C., Furey A., Jordan K. (2013). Investigation of the migration of triclabendazole residues to milk products manufactured from bovine milk, and stability therein, following lactating cow treatment. J. Dairy Sci..

[23] Dalton J.P., Robinson M.W., Mulcahy G., O’Neill S.M., Donnelly S. (2013). Immunomodulatory molecules of *Fasciola hepatica*: Candidates for both vaccine and immunotherapeutic development. Vet. Parasitol..

[24] Meemon K., Sobhon P. (2015). Juvenile-specific cathepsin proteases in *Fasciola* spp.: Their characteristics and vaccine efficacies. Parasitol. Res..

[25] McManus D.P. (2020). Recent Progress in the Development of Liver Fluke and Blood Fluke Vaccines. Vaccines.

[26] Dominguez M.F., González-Miguel J., Carmona C., Dalton J.P., Cwiklinski K., Tort J., Siles-Lucas M. (2018). Low allelic diversity in vaccine candidates genes from different locations sustain hope for *Fasciola hepatica* immunization. Vet. Parasitol..

[27] Harnett W. (2014). Secretory products of helminth parasites as immunomodulators. Mol. Biochem. Parasitol..

[28] Ryan S., Shiels J., Taggart C.C., Dalton J.P., Weldon S. (2020). *Fasciola hepatica*-Derived Molecules as Regulators of the Host Immune Response. Front. Immunol..

[29] Cwiklinski K., Dalton J.P. (2018). Advances in *Fasciola hepatica* research using ’omics’ technologies. Int. J. Parasitol..

[30] Corral-Ruiz G.M., Sánchez-Torres L.E. (2020). *Fasciola hepatica*-derived molecules as potential immunomodulators. Acta Trop..

[31] Cwiklinski K., Dalton J.P., Dufresne P.J., La Course J., Williams D.J., Hodgkinson J., Paterson S. (2015). The *Fasciola hepatica* genome: Gene duplication and polymorphism reveals adaptation to the host environment and the capacity for rapid evolution. Genome Biol..

[32] Robinson M.W., Tort J.F., Lowther J., Donnelly S.M., Wong E., Xu W., Stack C.M., Padula M., Herbert B., Dalton J.P. (2008). Proteomics and phylogenetic analysis of the cathepsin L protease family of the helminth pathogen *Fasciola hepatica*: Expansion of a repertoire of virulence-associated factors. Mol. Cell. Proteomics..

[33] Cwiklinski K., Donnelly S., Drysdale O., Jewhurst H., Smith D., De Marco Verissimo C., Pritsch I.C., O’Neill S., Dalton J.P., Robinson M.W. (2019). The cathepsin-like cysteine peptidases of trematodes of the genus *Fasciola*. Adv. Parasitol..

[34] González-Miguel J., Valero M.A., Reguera-Gomez M., Mas-Bargues C., Bargues M.D., Simón F., Mas-Coma S. (2019). Numerous *Fasciola* plasminogen-binding proteins may underlie blood-brain barrier leakage and explain neurological disorder complexity and heterogeneity in the acute and chronic phases of human fascioliasis. Parasitology.

[35] Hofmann B., Hecht H.J., Flohé L. (2002). Peroxiredoxins. Biol. Chem..

[36] Hayes J.D., Flanagan J.U., Jowsey I.R. (2005). Glutathione transferases. Annu. Rev. Pharmacol. Toxicol..

[37] Donnelly S., O’Neill S.M., Sekiya M., Mulcahy G., Dalton J.P. (2005). Thioredoxin peroxidase secreted by *Fasciola hepatica* induces the alternative activation of macrophages. Infect. Immun..

[38] Chemale G., Morphew R., Moxon J.V., Morassuti A.L., Lacourse E.J., Barrett J., Johnston D.A., Brophy P.M. (2006). Proteomic analysis of glutathione transferases from the liver fluke parasite, *Fasciola hepatica*. Proteomics.

[39] Spithill T.W., Smooker P.M., Copeman D.B., Dalton J.P. (1999). Fasciola gigantica: Epidemiology, control, immunology and molecular biology. Fasciolosis.

[40] Toh S.Q., Gobert G.N., Malagón Martínez D., Jones M.K. (2015). Haem uptake is essential for egg production in the haematophagous blood fluke of humans, *Schistosoma mansoni*. FEBS J..

[41] Costa-Paiva E.M., Coates C.J. (2020). Recent Insights into the Diversity and Evolution of Invertebrate Hemerythrins and Extracellular Globins. Subcell. Biochem..

[42] Dewilde S., Ioanitescu A.I., Kiger L., Gilany K., Marden M.C., Van Doorslaer S., Vercruysse J., Pesce A., Nardini M., Bolognesi M. (2008). The hemoglobins of the trematodes *Fasciola hepatica* and *Paramphistomum epiclitum*: A molecular biological, physico-chemical, kinetic, and vaccination study. Protein Sci..

[43] Cabán-Hernández K., Gaudier J.F., Espino A.M. (2012). Characterization and differential expression of a ferritin protein from *Fasciola hepatica*. Mol. Biochem. Parasitol..

[44] Jefferies J.R., Campbell A.M., van Rossum A.J., Barrett J., Brophy P.M. (2001). Proteomic analysis of *Fasciola hepatica* excretory-secretory products. Proteomics.

[45] Morphew R.M., Wright H.A., LaCourse E.J., Woods D.J., Brophy P.M. (2007). Comparative proteomics of excretory-secretory proteins released by the liver fluke *Fasciola hepatica* in sheep host bile and during in vitro culture ex host. Mol. Cell. Proteomics.

[46] Walsh T.R., Ainsworth S., Armstrong S., Hodgkinson J., Williams D. (2021). Differences in the antibody response to adult *Fasciola hepatica* excretory/secretory products in experimentally and naturally infected cattle and sheep. Vet. Parasitol..

[47] Shevchenko A., Wilm M., Vorm O., Mann M. (1996). Mass spectrometric sequencing of proteins silver-stained polyacrylamide gels. Anal. Chem..

