# Supplementary material for: Recognition Pattern of the Fasciola hepatica Excretome/Secretome during the Course of an Experimental Infection in Sheep by 2D Immunoproteomics
Source: Pathogens. 2021 Jun 9;10(6):725. doi: 10.3390/pathogens10060725 (PMC8228785; doi:10.3390/pathogens10060725)
Supplement: Supplementary file 1 [file pathogens-10-00725-s001.zip › Figure S2_R1.pdf]

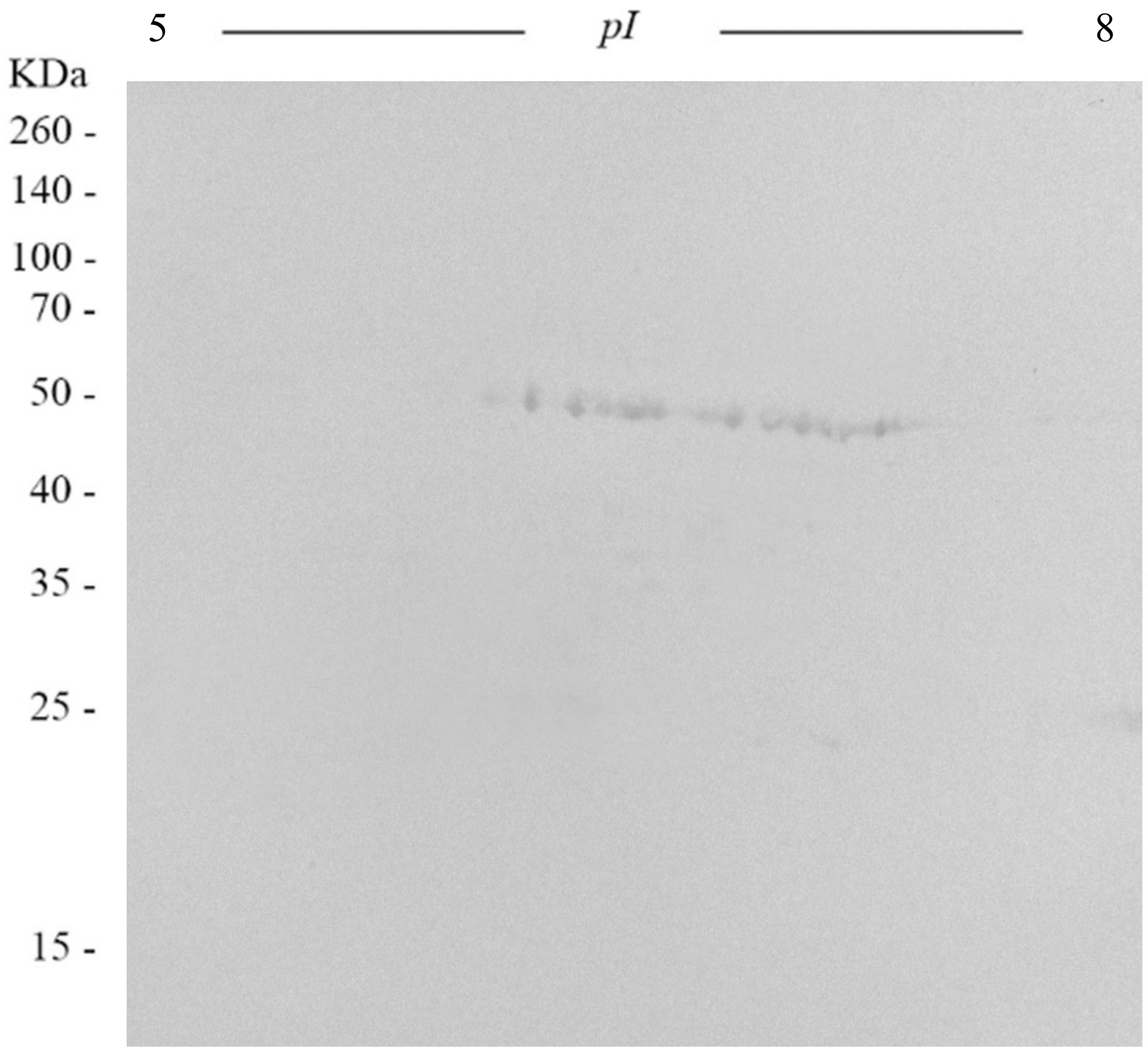

Figure S2. Representative western blot of the excretory/secretory products from adult worms of *F. hepatica* (FhES) revealed by a pool of serum samples from experimentally infected sheep before infection (control). The blot was in the 5–8 isoelectrical point range. Reference molecular weight masses are indicated on the left.
